# *N*-(2-Amino-3,5-dibromo­benz­yl)-*N*-methyl­cyclo­hexan-1-aminium *p*-toluenesulfonate

**DOI:** 10.1107/S1600536811027358

**Published:** 2011-07-13

**Authors:** Meenaxi M. Maste, Sudarshan Mahapatra, Krishna Kumar Ramachandran, K. N. Venugopala, A. R. Bhat

**Affiliations:** a Department of Pharmaceutical Chemistry, KLEU’s College of Pharmacy, Belgaum, India; b Solid State and Structural Chemistry Unit, Indian Institute of Science, Bangalore 560 012, India

## Abstract

The title compound, C_14_H_21_Br_2_N_2_^+^·C_7_H_7_O_3_S^−^, features a salt of protonated bromhexine, a pharmaceutical used in the treatment of respiratory disorders, and the *p*-toluenesulfonate anion. The crystal packing is stabilized by inter­molecular N—H⋯O, N—H⋯Br and C—H⋯O hydrogen bonds.

## Related literature

For salts of bromhexine, see: Koo *et al.* (1984 ▶); Shimizu & Nishigaki (1983 ▶); Shimizu *et al.* (1983 ▶, 1984 ▶).
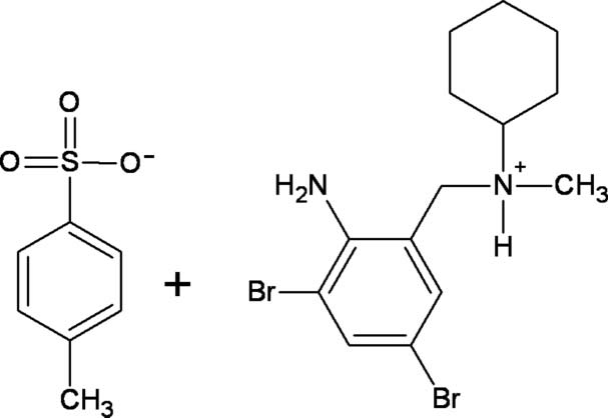


## Experimental

### 

#### Crystal data

C_14_H_21_Br_2_N_2_^+^·C_7_H_7_O_3_S^−^
*M* *_r_* = 548.32
Monoclinic, 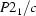

*a* = 14.008 (5) Å
*b* = 10.404 (5) Å
*c* = 17.157 (5) Å
β = 110.148 (5)°
*V* = 2347.4 (16) Å^3^
*Z* = 4
Mo *K*α radiation
μ = 3.57 mm^−1^
*T* = 293 K
0.30 × 0.10 × 0.08 mm

#### Data collection

Bruker SMART CCD area-detector diffractometer
Absorption correction: multi-scan (*SADABS*; Sheldrick, 1996 ▶) *T*_min_ = 0.414, *T*_max_ = 0.764
14496 measured reflections
4608 independent reflections
2343 reflections with *I* > 2σ(*I*)
*R*_int_ = 0.052

#### Refinement

*R*[*F* ^2^ > 2σ(*F* ^2^)] = 0.047
*wR*(*F* ^2^) = 0.114
*S* = 1.00
4608 reflections
272 parameters
4 restraints
H atoms treated by a mixture of independent and constrained refinement
Δρ_max_ = 0.78 e Å^−3^
Δρ_min_ = −0.71 e Å^−3^

### 

Data collection: *SMART* (Bruker, 1998 ▶); cell refinement: *SAINT* (Bruker, 1998 ▶); data reduction: *SAINT*; program(s) used to solve structure: *SHELXS97* (Sheldrick, 2008 ▶); program(s) used to refine structure: *SHELXL97* (Sheldrick, 2008 ▶); molecular graphics: *ORTEP-3 for Windows* (Farrugia, 1997 ▶); software used to prepare material for publication: *PLATON* (Spek, 2009 ▶).

## Supplementary Material

Crystal structure: contains datablock(s) global, I. DOI: 10.1107/S1600536811027358/bt5564sup1.cif

Supplementary material file. DOI: 10.1107/S1600536811027358/bt5564Isup2.mol

Structure factors: contains datablock(s) I. DOI: 10.1107/S1600536811027358/bt5564Isup3.hkl

Supplementary material file. DOI: 10.1107/S1600536811027358/bt5564Isup4.cml

Additional supplementary materials:  crystallographic information; 3D view; checkCIF report

## Figures and Tables

**Table 1:** Hydrogen-bond geometry (Å, °)

*D*—H⋯*A*	*D*—H	H⋯*A*	*D*⋯*A*	*D*—H⋯*A*
N1—H1⋯O2^i^	0.85 (3)	1.93 (4)	2.756 (4)	161 (4)
N2—H2*A*⋯O1^ii^	0.85 (3)	2.11 (3)	2.926 (4)	162 (4)
N2—H2*B*⋯Br1	0.84 (2)	2.67 (3)	3.068 (3)	111 (2)
C7—H7*A*⋯O2	0.97	2.47	3.257 (5)	138

Symmetry codes: (i) 
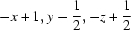
; (ii) 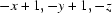.

**Fig. 1.**
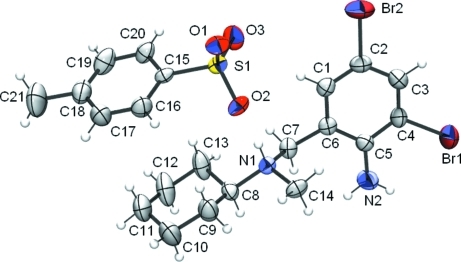
View of the title compound with the atom numbering scheme. Displacement ellipsoids for non-H atoms are drawn at the 50% probability level.

## supplementary crystallographic information

### Comment

There are only three crystal structures on Bromhexine reported in the literature. Analysis of all reported structure of Bromhexine indicates that the *N*-methyl amino group of Bromhexine is basic in nature and forms a salt with HCl (Koo *et al.*, 1984), salicylic acid (Shimizu *et al.*, 1984) and 1,2-benzisothiazol-3(2*H*)-one 1,1-dioxide (Shimizu *et al.*, 1983). A similar case is found in the current study where Bromhexine forms a salt with paratoluene sulfonic acid by transfering a proton from sulfonic acid group to *N*-methyl amino group. The crystal structure is stabilized by  N—H···O, N—H···Br and C—H···O intermolecular interactions.

### Experimental

An equimolar ratio (1:1) of Bromhexine and para-toluene sulfonic acid were dissolved in ethanol and kept for crystallization at room temperature yielding plate shape crystals.

### Refinement

In the absence of significant anomalous dispersion effects, Friedel pairs were merged. H1 was freely refined, but all other H atoms were positioned geometrically and refined using a riding model.

### Crystal data

**Table:**

C_14_H_21_Br_2_N_2_^+^·C_7_H_7_O_3_S^−^	*F*(000) = 1112
*M**_r_* = 548.32	*D*_x_ = 1.551 Mg m^−^^3^
Monoclinic, *P*2_1_/*c*	Mo *K*α radiation, λ = 0.71073 Å
Hall symbol: -P 2ybc	Cell parameters from 2343 reflections
*a* = 14.008 (5) Å	θ = 2.3–26.0°
*b* = 10.404 (5) Å	µ = 3.57 mm^−^^1^
*c* = 17.157 (5) Å	*T* = 293 K
β = 110.148 (5)°	Needle, colorless
*V* = 2347.4 (16) Å^3^	0.30 × 0.10 × 0.08 mm
*Z* = 4	

### Data collection

**Table:**

Bruker SMART CCD area-detector diffractometer	4608 independent reflections
Radiation source: fine-focus sealed tube	2343 reflections with *I* > 2σ(*I*)
graphite	*R*_int_ = 0.052
φ and ω scans	θ_max_ = 26.0°, θ_min_ = 2.3°
Absorption correction: multi-scan (*SADABS*; Sheldrick, 1996)	*h* = −15→17
*T*_min_ = 0.414, *T*_max_ = 0.764	*k* = −11→12
14496 measured reflections	*l* = −21→21

### Refinement

**Table:**

Refinement on *F*^2^	Primary atom site location: structure-invariant direct methods
Least-squares matrix: full	Secondary atom site location: difference Fourier map
*R*[*F*^2^ > 2σ(*F*^2^)] = 0.047	Hydrogen site location: inferred from neighbouring sites
*wR*(*F*^2^) = 0.114	H atoms treated by a mixture of independent and constrained refinement
*S* = 1.00	*w* = 1/[σ^2^(*F*_o_^2^) + (0.050*P*)^2^] where *P* = (*F*_o_^2^ + 2*F*_c_^2^)/3
4608 reflections	(Δ/σ)_max_ < 0.001
272 parameters	Δρ_max_ = 0.78 e Å^−^^3^
4 restraints	Δρ_min_ = −0.71 e Å^−^^3^

### Special details

**Table:**

Geometry. Bond distances, angles *etc*. have been calculated using the rounded fractional coordinates. All su's are estimated from the variances of the (full) variance-covariance matrix. The cell e.s.d.'s are taken into account in the estimation of distances, angles and torsion angles
Refinement. Refinement of *F*^2^ against ALL reflections. The weighted *R*-factor *wR* and goodness of fit *S* are based on *F*^2^, conventional *R*-factors *R* are based on *F*, with *F* set to zero for negative *F*^2^. The threshold expression of *F*^2^ > 2σ(*F*^2^) is used only for calculating *R*-factors(gt) *etc*. and is not relevant to the choice of reflections for refinement. *R*-factors based on *F*^2^ are statistically about twice as large as those based on *F*, and *R*- factors based on ALL data will be even larger.

### Fractional atomic coordinates and isotropic or equivalent isotropic displacement parameters (Å^2^)

**Table:**

	*x*	*y*	*z*	*U*_iso_*/*U*_eq_	
Br1	0.06429 (3)	0.35949 (7)	−0.08435 (3)	0.0854 (3)	
Br2	0.14386 (4)	0.34597 (7)	0.25939 (3)	0.0893 (3)	
N1	0.5129 (2)	0.2898 (3)	0.1723 (2)	0.0330 (11)	
N2	0.2969 (2)	0.3732 (5)	−0.0301 (2)	0.0666 (16)	
C1	0.2959 (3)	0.3727 (4)	0.1844 (2)	0.0411 (14)	
C2	0.1945 (3)	0.3605 (4)	0.1704 (2)	0.0500 (16)	
C3	0.1249 (3)	0.3565 (4)	0.0899 (3)	0.0548 (18)	
C4	0.1613 (3)	0.3629 (4)	0.0259 (2)	0.0466 (14)	
C5	0.2651 (3)	0.3701 (4)	0.0360 (2)	0.0411 (14)	
C6	0.3321 (3)	0.3795 (4)	0.1181 (2)	0.0356 (12)	
C7	0.4449 (3)	0.4040 (4)	0.1372 (2)	0.0406 (14)	
C8	0.6233 (3)	0.3192 (4)	0.1819 (2)	0.0424 (14)	
C9	0.6906 (3)	0.2040 (5)	0.2153 (3)	0.0628 (16)	
C10	0.8014 (3)	0.2361 (6)	0.2280 (3)	0.095 (3)	
C11	0.8394 (3)	0.3534 (5)	0.2807 (3)	0.080 (2)	
C12	0.7710 (3)	0.4670 (5)	0.2473 (3)	0.084 (2)	
C13	0.6605 (3)	0.4379 (4)	0.2365 (3)	0.0639 (18)	
C14	0.4737 (3)	0.1716 (4)	0.1239 (2)	0.0419 (14)	
S1	0.48360 (7)	0.75893 (10)	0.09405 (5)	0.0358 (3)	
O1	0.50153 (19)	0.6560 (3)	0.04343 (15)	0.0440 (10)	
O2	0.48544 (18)	0.7100 (3)	0.17409 (14)	0.0436 (9)	
O3	0.5480 (2)	0.8695 (3)	0.10102 (18)	0.0572 (11)	
C15	0.3570 (3)	0.8101 (4)	0.0421 (2)	0.0317 (11)	
C16	0.2762 (3)	0.7309 (4)	0.0396 (2)	0.0486 (16)	
C17	0.1786 (3)	0.7695 (5)	0.0001 (3)	0.0632 (19)	
C18	0.1571 (3)	0.8892 (5)	−0.0385 (3)	0.0596 (19)	
C19	0.2376 (3)	0.9666 (5)	−0.0353 (2)	0.0599 (17)	
C20	0.3377 (3)	0.9273 (4)	0.0037 (2)	0.0455 (16)	
C21	0.0474 (3)	0.9326 (6)	−0.0849 (3)	0.097 (2)	
H1	0.510 (3)	0.283 (4)	0.221 (2)	0.039 (12)*	
H1A	0.34145	0.37656	0.23856	0.0493*	
H2A	0.3594 (16)	0.376 (5)	−0.025 (2)	0.1165*	
H2B	0.257 (2)	0.362 (5)	−0.0791 (13)	0.1165*	
H3	0.05550	0.34954	0.08003	0.0656*	
H7A	0.46572	0.47432	0.17655	0.0483*	
H7B	0.45510	0.43113	0.08657	0.0483*	
H8	0.62560	0.33869	0.12668	0.0509*	
H9A	0.68487	0.17775	0.26774	0.0753*	
H9B	0.66849	0.13292	0.17664	0.0753*	
H10A	0.80824	0.24959	0.17422	0.1137*	
H10B	0.84369	0.16334	0.25395	0.1137*	
H11A	0.84262	0.33587	0.33704	0.0963*	
H11B	0.90757	0.37357	0.28204	0.0963*	
H12A	0.79431	0.53895	0.28507	0.1008*	
H12B	0.77540	0.49181	0.19414	0.1008*	
H13A	0.65444	0.42325	0.29040	0.0774*	
H13B	0.61841	0.51114	0.21117	0.0774*	
H14A	0.51760	0.10072	0.14848	0.0629*	
H14B	0.40641	0.15390	0.12394	0.0629*	
H14C	0.47148	0.18343	0.06781	0.0629*	
H16	0.28896	0.65083	0.06524	0.0582*	
H17	0.12543	0.71510	−0.00111	0.0757*	
H19	0.22494	1.04752	−0.05974	0.0719*	
H20	0.39114	0.98030	0.00355	0.0546*	
H21A	0.00115	0.86721	−0.08053	0.1456*	
H21B	0.03425	1.01093	−0.06074	0.1456*	
H21C	0.03824	0.94686	−0.14227	0.1456*	

### Atomic displacement parameters (Å^2^)

**Table:**

	*U*^11^	*U*^22^	*U*^33^	*U*^12^	*U*^13^	*U*^23^
Br1	0.0448 (3)	0.1514 (7)	0.0473 (3)	0.0073 (3)	−0.0002 (2)	−0.0107 (3)
Br2	0.0855 (4)	0.1380 (7)	0.0625 (3)	0.0055 (4)	0.0488 (3)	−0.0046 (3)
N1	0.0363 (18)	0.030 (2)	0.0331 (18)	−0.0035 (16)	0.0126 (15)	−0.0005 (17)
N2	0.049 (2)	0.114 (4)	0.040 (2)	0.006 (3)	0.0193 (17)	0.007 (2)
C1	0.043 (2)	0.039 (3)	0.038 (2)	0.002 (2)	0.0096 (18)	−0.001 (2)
C2	0.053 (3)	0.061 (3)	0.042 (2)	0.001 (3)	0.024 (2)	−0.002 (2)
C3	0.040 (2)	0.073 (4)	0.053 (3)	−0.001 (2)	0.018 (2)	−0.006 (3)
C4	0.035 (2)	0.059 (3)	0.039 (2)	0.005 (2)	0.0042 (18)	−0.001 (2)
C5	0.038 (2)	0.046 (3)	0.039 (2)	0.006 (2)	0.0130 (18)	−0.002 (2)
C6	0.039 (2)	0.026 (2)	0.041 (2)	0.001 (2)	0.0128 (18)	0.001 (2)
C7	0.039 (2)	0.034 (3)	0.044 (2)	−0.001 (2)	0.0081 (18)	0.004 (2)
C8	0.032 (2)	0.053 (3)	0.042 (2)	−0.004 (2)	0.0124 (17)	0.003 (2)
C9	0.045 (2)	0.062 (3)	0.072 (3)	0.009 (3)	0.008 (2)	−0.017 (3)
C10	0.045 (3)	0.124 (6)	0.107 (4)	0.011 (4)	0.016 (3)	−0.038 (4)
C11	0.041 (3)	0.110 (5)	0.080 (4)	−0.014 (3)	0.008 (2)	−0.016 (4)
C12	0.046 (3)	0.084 (4)	0.101 (4)	−0.016 (3)	−0.002 (3)	0.018 (4)
C13	0.040 (2)	0.039 (3)	0.096 (4)	−0.005 (2)	0.002 (2)	0.003 (3)
C14	0.059 (2)	0.026 (3)	0.042 (2)	−0.004 (2)	0.019 (2)	−0.008 (2)
S1	0.0422 (5)	0.0333 (7)	0.0320 (5)	−0.0031 (6)	0.0131 (4)	0.0001 (5)
O1	0.0552 (16)	0.044 (2)	0.0405 (15)	0.0049 (14)	0.0263 (13)	−0.0070 (14)
O2	0.0583 (16)	0.0459 (19)	0.0260 (13)	0.0045 (15)	0.0137 (12)	0.0001 (13)
O3	0.0478 (16)	0.045 (2)	0.070 (2)	−0.0120 (16)	0.0089 (14)	0.0067 (16)
C15	0.043 (2)	0.028 (2)	0.0253 (18)	−0.002 (2)	0.0133 (16)	−0.0019 (19)
C16	0.051 (3)	0.041 (3)	0.051 (2)	−0.002 (3)	0.014 (2)	0.012 (2)
C17	0.045 (3)	0.079 (4)	0.062 (3)	−0.005 (3)	0.014 (2)	0.016 (3)
C18	0.050 (3)	0.078 (4)	0.045 (3)	0.012 (3)	0.009 (2)	0.001 (3)
C19	0.076 (3)	0.039 (3)	0.052 (3)	0.009 (3)	0.006 (2)	0.003 (2)
C20	0.053 (3)	0.040 (3)	0.035 (2)	−0.005 (2)	0.0042 (19)	−0.001 (2)
C21	0.059 (3)	0.114 (5)	0.096 (4)	0.027 (3)	−0.002 (3)	0.012 (4)

### Geometric parameters (Å, °)

**Table:**

Br1—C4	1.912 (4)	C7—H7B	0.9700
Br2—C2	1.898 (4)	C8—H8	0.9800
S1—O1	1.454 (3)	C9—H9B	0.9700
S1—O2	1.456 (3)	C9—H9A	0.9700
S1—O3	1.441 (3)	C10—H10B	0.9700
S1—C15	1.770 (4)	C10—H10A	0.9700
N1—C7	1.512 (5)	C11—H11A	0.9700
N1—C8	1.529 (5)	C11—H11B	0.9700
N1—C14	1.480 (5)	C12—H12A	0.9700
N2—C5	1.354 (5)	C12—H12B	0.9700
N1—H1	0.85 (3)	C13—H13A	0.9700
N2—H2B	0.84 (2)	C13—H13B	0.9700
N2—H2A	0.85 (3)	C14—H14B	0.9600
C1—C2	1.363 (6)	C14—H14C	0.9600
C1—C6	1.398 (5)	C14—H14A	0.9600
C2—C3	1.390 (6)	C15—C16	1.389 (6)
C3—C4	1.362 (6)	C15—C20	1.368 (6)
C4—C5	1.406 (6)	C16—C17	1.361 (6)
C5—C6	1.401 (5)	C17—C18	1.394 (7)
C6—C7	1.521 (6)	C18—C19	1.371 (7)
C8—C9	1.511 (7)	C18—C21	1.535 (7)
C8—C13	1.529 (6)	C19—C20	1.391 (6)
C9—C10	1.528 (7)	C16—H16	0.9300
C10—C11	1.503 (8)	C17—H17	0.9300
C11—C12	1.505 (7)	C19—H19	0.9300
C12—C13	1.525 (7)	C20—H20	0.9300
C1—H1A	0.9300	C21—H21A	0.9600
C3—H3	0.9300	C21—H21B	0.9600
C7—H7A	0.9700	C21—H21C	0.9600
			
Br1···N2	3.068 (3)	H1A···H7A	2.5400
Br1···Br2^i^	3.880 (2)	H1A···O3^viii^	2.6500
Br2···Br1^ii^	3.880 (2)	H2A···H7B	2.0100
Br1···H2B	2.67 (3)	H2A···S1^iv^	3.16 (3)
Br2···H19^iii^	3.1200	H2A···O1^iv^	2.11 (3)
S1···H14C^iv^	3.1100	H2A···C7	2.64 (3)
S1···H1^v^	3.15 (3)	H2B···Br1	2.67 (3)
S1···H2A^iv^	3.16 (3)	H2B···H12B^iv^	2.4100
O1···C7	3.312 (5)	H3···H17^x^	2.5200
O1···N2^iv^	2.926 (4)	H3···H21A^x^	2.3900
O2···N1^v^	2.756 (4)	H7A···H1A	2.5400
O2···C14^v^	3.337 (4)	H7A···C13	2.5900
O2···C7	3.257 (5)	H7A···H13B	2.0500
O3···C14^vi^	3.376 (5)	H7A···O2	2.4700
O1···H2A^iv^	2.11 (3)	H7B···N2	2.5000
O1···H8^iv^	2.8500	H7B···O1	2.6000
O1···H7B	2.6000	H7B···H8	2.4400
O1···H7B^iv^	2.6600	H7B···O1^iv^	2.6600
O1···H14C^iv^	2.6600	H7B···H2A	2.0100
O2···H13B	2.7100	H8···H12B	2.5700
O2···H16	2.8100	H8···O1^iv^	2.8500
O2···H7A	2.4700	H8···H7B	2.4400
O2···H9A^v^	2.9100	H8···H10A	2.5800
O2···H1^v^	1.93 (4)	H9A···O2^viii^	2.9100
O3···H20	2.5300	H9A···H1	2.5500
O3···H1A^v^	2.6500	H9B···C14	2.5900
O3···H14C^iv^	2.8700	H9B···H14A	2.0300
O3···H20^vii^	2.7300	H10A···C18^iv^	2.9200
O3···H14A^vi^	2.6200	H10A···C17^iv^	3.0700
N1···O2^viii^	2.756 (4)	H10A···H8	2.5800
N2···Br1	3.068 (3)	H11A···C17^viii^	2.9900
N2···O1^iv^	2.926 (4)	H12B···H2B^iv^	2.4100
N2···H7B	2.5000	H12B···H8	2.5700
C5···C14	3.470 (6)	H13A···H1	2.4500
C7···O2	3.257 (5)	H13B···H7A	2.0500
C7···O1	3.312 (5)	H13B···O2	2.7100
C14···C20^ix^	3.411 (6)	H13B···C7	2.5800
C14···O2^viii^	3.337 (4)	H14A···O3^ix^	2.6200
C14···O3^ix^	3.376 (5)	H14A···H9B	2.0300
C14···C5	3.470 (6)	H14A···C9	2.5400
C20···C14^vi^	3.411 (6)	H14B···C5	3.0300
C1···H1	2.99 (4)	H14B···C20^ix^	3.0600
C3···H21A^x^	2.8900	H14B···C6	2.5600
C5···H16	2.9600	H14C···O3^iv^	2.8700
C5···H14B	3.0300	H14C···O1^iv^	2.6600
C6···H16	2.9600	H14C···H20^ix^	2.4700
C6···H14B	2.5600	H14C···S1^iv^	3.1100
C7···H2A	2.64 (3)	H16···O2	2.8100
C7···H13B	2.5800	H16···C5	2.9600
C9···H14A	2.5400	H16···C6	2.9600
C13···H7A	2.5900	H17···H21A	2.4000
C14···H20^ix^	2.8100	H17···H3^x^	2.5200
C14···H9B	2.5900	H19···Br2^xi^	3.1200
C17···H11A^v^	2.9900	H20···O3	2.5300
C17···H10A^iv^	3.0700	H20···C14^vi^	2.8100
C18···H10A^iv^	2.9200	H20···H14C^vi^	2.4700
C20···H14B^vi^	3.0600	H20···O3^vii^	2.7300
H1···H13A	2.4500	H21A···H17	2.4000
H1···S1^viii^	3.15 (3)	H21A···C3^x^	2.8900
H1···H9A	2.5500	H21A···H3^x^	2.3900
H1···C1	2.99 (4)	H21B···H21B^xii^	2.5900
H1···O2^viii^	1.93 (4)		
			
O2—S1—O3	113.17 (17)	C8—C9—H9A	110.00
O2—S1—C15	105.61 (17)	C10—C9—H9B	109.00
O3—S1—C15	106.95 (19)	C11—C10—H10A	109.00
O1—S1—O3	113.92 (18)	C11—C10—H10B	109.00
O1—S1—C15	105.66 (17)	C9—C10—H10B	109.00
O1—S1—O2	110.80 (17)	C9—C10—H10A	109.00
C8—N1—C14	113.1 (3)	H10A—C10—H10B	108.00
C7—N1—C8	111.2 (3)	C10—C11—H11B	109.00
C7—N1—C14	111.6 (3)	C10—C11—H11A	109.00
C7—N1—H1	103 (3)	H11A—C11—H11B	108.00
C8—N1—H1	107 (3)	C12—C11—H11A	109.00
C14—N1—H1	110 (3)	C12—C11—H11B	109.00
C5—N2—H2A	123 (2)	C11—C12—H12A	109.00
H2A—N2—H2B	114 (3)	C13—C12—H12A	109.00
C5—N2—H2B	122 (2)	C13—C12—H12B	109.00
C2—C1—C6	120.6 (3)	C11—C12—H12B	109.00
Br2—C2—C3	117.9 (3)	H12A—C12—H12B	108.00
Br2—C2—C1	121.4 (3)	C12—C13—H13A	110.00
C1—C2—C3	120.7 (4)	C12—C13—H13B	109.00
C2—C3—C4	118.1 (4)	C8—C13—H13A	110.00
Br1—C4—C3	117.4 (3)	C8—C13—H13B	109.00
C3—C4—C5	124.2 (3)	H13A—C13—H13B	108.00
Br1—C4—C5	118.4 (3)	H14B—C14—H14C	109.00
N2—C5—C4	121.5 (3)	N1—C14—H14A	110.00
N2—C5—C6	122.7 (4)	N1—C14—H14B	109.00
C4—C5—C6	115.7 (3)	N1—C14—H14C	109.00
C1—C6—C5	120.6 (4)	H14A—C14—H14B	109.00
C5—C6—C7	121.0 (3)	H14A—C14—H14C	109.00
C1—C6—C7	118.3 (3)	S1—C15—C16	120.1 (3)
N1—C7—C6	114.8 (3)	S1—C15—C20	120.5 (3)
C9—C8—C13	111.8 (3)	C16—C15—C20	119.4 (4)
N1—C8—C9	111.0 (3)	C15—C16—C17	120.6 (4)
N1—C8—C13	110.3 (3)	C16—C17—C18	121.1 (4)
C8—C9—C10	110.6 (4)	C17—C18—C19	117.7 (4)
C9—C10—C11	113.3 (4)	C17—C18—C21	121.5 (4)
C10—C11—C12	111.2 (4)	C19—C18—C21	120.7 (5)
C11—C12—C13	112.1 (4)	C18—C19—C20	121.7 (4)
C8—C13—C12	110.6 (4)	C15—C20—C19	119.5 (4)
C6—C1—H1A	120.00	C15—C16—H16	120.00
C2—C1—H1A	120.00	C17—C16—H16	120.00
C2—C3—H3	121.00	C16—C17—H17	119.00
C4—C3—H3	121.00	C18—C17—H17	120.00
N1—C7—H7A	109.00	C18—C19—H19	119.00
C6—C7—H7B	109.00	C20—C19—H19	119.00
N1—C7—H7B	109.00	C15—C20—H20	120.00
C6—C7—H7A	109.00	C19—C20—H20	120.00
H7A—C7—H7B	108.00	C18—C21—H21A	109.00
C13—C8—H8	108.00	C18—C21—H21B	109.00
N1—C8—H8	108.00	C18—C21—H21C	110.00
C9—C8—H8	108.00	H21A—C21—H21B	109.00
H9A—C9—H9B	108.00	H21A—C21—H21C	110.00
C8—C9—H9B	110.00	H21B—C21—H21C	110.00
C10—C9—H9A	110.00		
			
O2—S1—C15—C16	−47.4 (3)	N2—C5—C6—C1	−178.4 (4)
O1—S1—C15—C16	70.1 (3)	N2—C5—C6—C7	4.7 (7)
O1—S1—C15—C20	−109.6 (3)	C4—C5—C6—C7	−172.6 (4)
O3—S1—C15—C20	12.1 (4)	C4—C5—C6—C1	4.3 (6)
O2—S1—C15—C20	133.0 (3)	C1—C6—C7—N1	75.0 (5)
O3—S1—C15—C16	−168.2 (3)	C5—C6—C7—N1	−108.1 (4)
C8—N1—C7—C6	174.7 (3)	N1—C8—C9—C10	−177.7 (3)
C14—N1—C8—C13	−176.8 (3)	C9—C8—C13—C12	55.3 (5)
C7—N1—C8—C9	−178.8 (3)	C13—C8—C9—C10	−54.1 (5)
C7—N1—C8—C13	56.7 (4)	N1—C8—C13—C12	179.3 (3)
C14—N1—C7—C6	47.3 (4)	C8—C9—C10—C11	53.7 (5)
C14—N1—C8—C9	−52.3 (4)	C9—C10—C11—C12	−53.7 (6)
C2—C1—C6—C5	−1.9 (6)	C10—C11—C12—C13	54.2 (5)
C2—C1—C6—C7	175.1 (4)	C11—C12—C13—C8	−55.1 (5)
C6—C1—C2—C3	−1.0 (6)	S1—C15—C16—C17	179.8 (3)
C6—C1—C2—Br2	178.1 (3)	C20—C15—C16—C17	−0.6 (6)
Br2—C2—C3—C4	−178.0 (3)	S1—C15—C20—C19	−178.7 (3)
C1—C2—C3—C4	1.1 (6)	C16—C15—C20—C19	1.7 (5)
C2—C3—C4—Br1	−179.2 (3)	C15—C16—C17—C18	−0.2 (7)
C2—C3—C4—C5	1.7 (6)	C16—C17—C18—C19	0.0 (7)
C3—C4—C5—N2	178.4 (4)	C16—C17—C18—C21	178.6 (4)
Br1—C4—C5—N2	−0.7 (6)	C17—C18—C19—C20	1.1 (6)
Br1—C4—C5—C6	176.6 (3)	C21—C18—C19—C20	−177.6 (4)
C3—C4—C5—C6	−4.3 (6)	C18—C19—C20—C15	−1.9 (6)

Symmetry codes: (i) *x*, −*y*+1/2, *z*−1/2; (ii) *x*, −*y*+1/2, *z*+1/2; (iii) *x*, −*y*+3/2, *z*+1/2; (iv) −*x*+1, −*y*+1, −*z*; (v) −*x*+1, *y*+1/2, −*z*+1/2; (vi) *x*, *y*+1, *z*; (vii) −*x*+1, −*y*+2, −*z*; (viii) −*x*+1, *y*−1/2, −*z*+1/2; (ix) *x*, *y*−1, *z*; (x) −*x*, −*y*+1, −*z*; (xi) *x*, −*y*+3/2, *z*−1/2; (xii) −*x*, −*y*+2, −*z*.

### Hydrogen-bond geometry (Å, °)

**Table:**

*D*—H···*A*	*D*—H	H···*A*	*D*···*A*	*D*—H···*A*
N1—H1···O2^viii^	0.85 (3)	1.93 (4)	2.756 (4)	161 (4)
N2—H2A···O1^iv^	0.85 (3)	2.11 (3)	2.926 (4)	162 (4)
N2—H2B···Br1	0.84 (2)	2.67 (3)	3.068 (3)	111 (2)
C7—H7A···O2	0.9700	2.4700	3.257 (5)	138.00
C20—H20···O3	0.9300	2.5300	2.905 (5)	104.00

Symmetry codes: (viii) −*x*+1, *y*−1/2, −*z*+1/2; (iv) −*x*+1, −*y*+1, −*z*.
